# Modes of Neighbouring Group Participation by the Methyl Selenyl Substituent in β-Methylselenylmethyl-substituted 1-Phenylethyl Carbenium Ions

**DOI:** 10.3390/molecules181011705

**Published:** 2013-09-25

**Authors:** Benjamin L. Harris, Jonathan M. White

**Affiliations:** School of Chemistry and BIO-21 Institute, University of Melbourne, Parkville 3010, Victoria, Australia; E-Mail: b.harris@student.unimelb.edu.au

**Keywords:** hyperconjugation, stereoelectronic effects, selenium interactions

## Abstract

Selenium substituents which are disposed β to an electron deficient centre, such as a carbocation p-orbital, or the π* orbital of an electron deficient p-system, interact in a stabilising way by a combination of C-Se hyperconjugation (σ_Se-C_–π* interaction), and a through-space homoconjugative n_Se_–π* interaction. The relative importance of these two modes of interaction is dependant on the electron demand of the cation, with hyperconjugation predominating for low electron demand systems, and the n_Se_–π* interaction predominating for high electron demand cations.

## 1. Introduction

**Figure 1:**
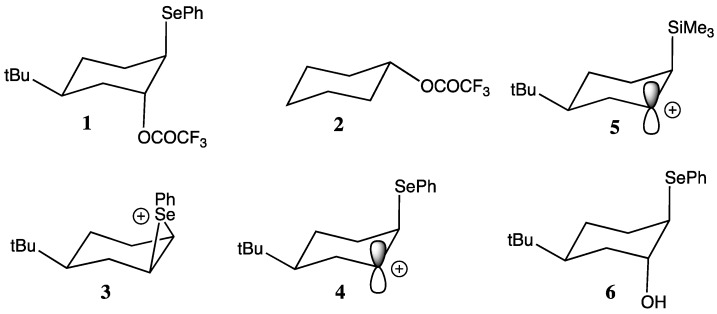
Modes of participation by β-Selenium substituents.

Unimolecular solvolyses of the conformationally biased β-phenylselenyl trifluoroacetate **1** (Figure 1) occurs at a rate which is 10^7^ times faster than the corresponding unsubstituted derivative **2** (Figure 1) suggesting that the selenium substituent provides strong assistance in the departure of the trifluoroacetate leaving group [1]. The mechanism of participation by the selenium substituent might reasonably be described by conventional neighbouring group participation [2]. In this case the selenium lone pair electrons act as an internal nucleophile, displacing the leaving group to give the seleniranium ion intermediate **3** (Figure 1). This is an example of non-vertical participation [3].

**Figure 2:**
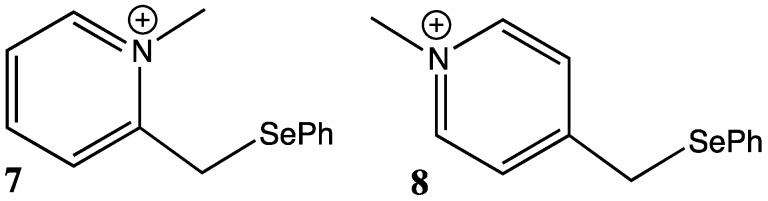
Selenium substituted pyridinium ions.

**Figure 3:**
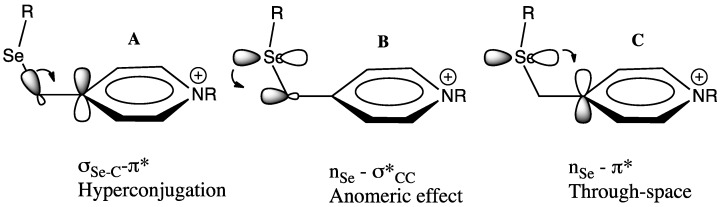
Orbital interactions involving the selenium substituent in 2- and 4-substituted pyridinium ions.

However, consideration was given to the possibility, that participation by the selenium substituent might occur by σ_C-Se_-p hyperconjugation (vertical participation), and involve the open carbenium ion **4** (Figure 1) as an intermediate [3,4,5,6]. This mode of participation is analogous to that provided by the trimethylsilyl substituent in the carbenium ion **5** (Figure 1) which is the basis of the silicon β-effect [7,8,9,10]. Application of the variable oxygen probe to ether and ester derivatives of the antiperiplanar β-phenylselenyl alcohol **6** (Figure 1) provided crystallographic evidence that the C-Se bond is a strong σ-donor and can therefore effectively stabilise a neighbouring carbenium ion by hyperconjugation alone [1]. More recently NMR, crystallographic and computational studies on phenylselenylmethyl-substituted pyridinium ions **7** and **8** (Figure 2) revealed that a number of orbital interactions involving the selenium substituent were responsible for stabilisation of the charge on the adjacent carbon (Figure 3) [11].

The orbital interactions include σ_Se-C_-π* hyperconjugation (Figure 3A), an anomeric effect (Figure 3B) and a through-space interaction between the selenium p-type lone pair orbital and the electron deficient pyridinium ion π system (Figure 3C) which represents the early stages of the bridging interaction as represented by structure **3** above. The latter two interactions explain the preferred gauche dihedral angle about the Se-C bond in these structures, a conformation, which is also preferred in α-phenylselenyl ketones, where similar orbital interactions are plausible [12,13].

**Figure 4:**
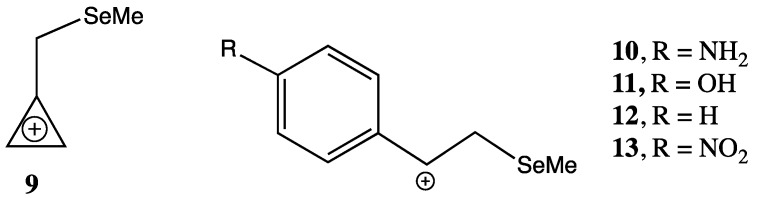
β-Selenium-substituted ions with higher electron demand.

Calculations showed that C-Se hyperconjugation (σ_C-Se_–π*) is the predominant mode of stabilisation in the weakly electron demanding pyridinium ions **7** and **8**, where the σ_C-Se_–π* hyperconjugative interaction provides 34.8 and 34.2 kJ mol^−1^ stabilisation respectively, while the through-space n_Se_–π* interaction provides 9.0 and 8.0 kJ mol^−1^ of stabilisation. However the through-space (n_Se_–π*) interaction becomes more important as the electron demand of the β-cation increases. For example, in the selenylmethyl-substituted cyclopropenium ion **9** the NBO interaction energies for the σ_C-Se_–π* interaction is 104.9 kJ mol^−1^ while the n_Se_–π* through-space interaction is 73.7 kJ mol^-1^. Also consistent with the increasing importance of the through space interaction as the electron demand increases is the closing of the Se-C-C(+) bond angle, which decreases from 110.9° for the pyridinium ion **8** to 101.1° in the cyclopropenium ion **9**. The anomeric interaction (n_Se_–σ*_CC_) was found to be relatively unimportant in all ions. In this paper we investigate computationally the relative importance of the stabilising orbital interactions in the more highly electron demanding ions **10**–**13** (Figure 4).

## 2. Methods

Calculations were performed at the B3LYP/6-311++G** level of theory [14,15,16,17,18], a level of theory which has been previously employed to investigate stereoelectronic effects of chalcogen substituents [11,19]. Natural Bond Orbitals (NBOs) were calculated using the NBO 3.1 program [20] as implemented in the Gaussian 03 package [21].

## 3. Results and Discussion

**Figure 5:**
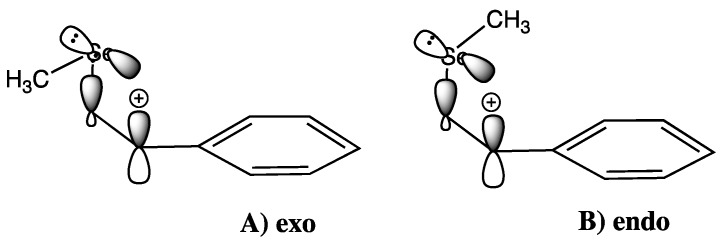
Low energy conformations of the seleniranium ion **12**.

The parent benzylically-stabilised *β*-selenium substituted 1-phenylethyl cation **12** has two low energy conformations, both of which allow vertical and non-vertical modes of participation to occur. In both conformations the C-Se bond is aligned with the direction of the carbenium ion p-orbital, allowing for σ_C-Se_–π* hyperconjugation to occur effectively, in addition the CH_3_-Se-CH_2_-C(+) dihedral angle is close to orthogonal, which allows the through-space n_Se_–π* interaction between the selenium p-type lone pair orbital and the carbenium ion p-orbital to occur. This gives rise to the exo conformation **12a** and the endo conformation **12b** these conformations are very similar energetically, with the exo conformer being slightly favoured (1.1 kJ mol^−1^) (Figure 5). For practical purposes the comparisons made below apply to the exo conformer.

**Figure 6:**
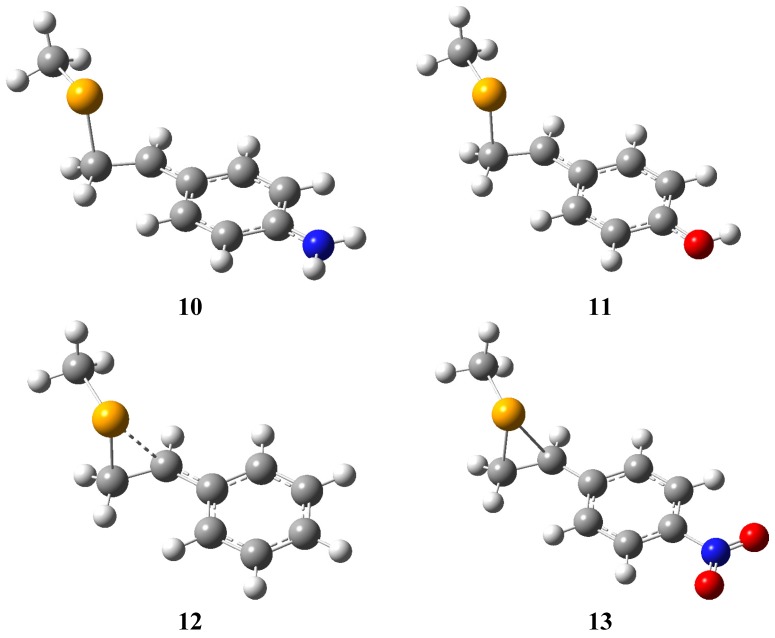
Calculated structures for the *β*-selenium substituted 1-phenylethyl cations **10**–**13**.

The computed structures of the β-selenyl-carbenium ions **10**–**13** are presented in Figure 6, while selected geometrical parameters and NBO orbital interaction energies are presented in Table 1 [20]. A convenient measure of electron demand of a cation is the pK_R_+ value, those, which are available from the literature have been included in this table.

Decreasing stabilisation of the carbenium ions by delocalization into the aromatic ring is demonstrated by the C(Ar)-C^+^ distance which increases from 1.389 Å in **10** to 1.452 Å in **13** where there is little resonance interaction. The general trend apparent from Table 1 is that as the magnitude of both σ_C-Se_–π hyperconjugation and the through-space n_Se_–p interaction increases with increasing electron demand of the 1-phenylethyl cation. Increasing strength of σ_C-Se_–π hyperconjugation is evident from the decreasing population of the σ_C-Se_ orbital with increasing electron demand, and the increasing magnitude of the orbital overlap term [F(i,j)], while the increasing strength of the through-space n_Se_-p interaction is evident from the decreasing population of the n_Se_ p-type lone pair orbital with increasing electron demand, and an increasing orbital overlap term [F(I,j)]. However the relative importance of the through-space stabilising interaction increases with increasing electron demand. For example in the relatively stable 4-amino-1-phenylethyl cation **10** σ_C-Se_–π hyperconjugation is the most important stabilising interaction (55.7 kJ mol^−1^ *vs.* 17.2 kJ mol^−1^ involving the selenium substituent, however while this stabilising interaction increases with increasing electron demand, the n_Se_–p through space interaction increases more profoundly, and in the parent cation **12** the through space through-space n_Se_–p interaction is the most important stabilising interaction (418.4 *vs.* 211.6 kJ mol^−1^). The increasing importance of the through-space interaction is consistent with the steady closing of the Se-CH_2_-C(+) bond angle from **10**–**12**, while in the most electron deficient cation, the *p*-nitrophenylethyl cation the ion is bridged, and the individual contributions from hyperconjugation and the through-space interaction can no-longer be deconvoluted.

**Table 1:** Structural and orbital properties, and NBO interaction energies of *β*-selenium substituted 1-phenylethyl cations **10**–**13**.

	10	11	12	13
Se-CH_2_ (Å)	2.012	2.011	2.010	2.010
Se-CH_2_-C+ (°)	98.84	93.41	84.78	78.96
Se…C(+) Å	2.664	2.554	2.372	2.245
C(Ar)-C+	1.389	1.403	1.423	1.452
pK_R_+ ^a^		−12.4 [ 22]	<−20 [ 23]	
Vertical interaction E(2) (kJ mol^−1^)	55.7	73.3	211.6	-
σC-Seenergy (a.u.)	−0.641	−0.653	−0.660	
σC-Sepopulation	1.891	1.866	1.820	
Overlap, F(i,j) (a.u.)	0.076	0.087	0.139	
Nonvertical interaction E(2) (kJ mol^−1^)	17.2	49.0	418.4	-
*n*Seenergy (a.u.)	−0.363	−0.379	−0.397	
*n*Sepopulation	1.799	1.727	1.606	
F(i,j) (a.u.)	0.037	0.046	0.083	

^a^ pK_R_+ values for the corresponding non-substituted carbenium ions.

## 4. Conclusions

Selenium substituents interact with electron deficient orbitals at the β-position by a combination of C-Se hyperconjugation, in which the electrons in the σ_C-Se_ bonding orbital mix with the electron deficient orbital, and a through space interaction between the selenium p-type lone pair orbital and the electron deficient centre, this latter interaction is also referred to as homo-conjugation. For cations with low electron demand, there is very little distortion of the Se-C-C(+) bond angle, and the most important mode of stabilisation is by σ_C-Se_–π hyperconjugation. However as the electron demand of the cation increases, then closing of the Se-C-C(+) bond angle occurs, this increases the orbital overlap between the selenium p-type lone pair orbital and the cabocation p-orbital and this becomes the predominant mode of stabilisation.

## Supplementary Materials

Supplementary materials can be accessed at: http://www.mdpi.com/1420-3049/18/10/11705/s1.
