# Supplementary material for: Modes of Neighbouring Group Participation by the Methyl Selenyl Substituent in β-Methylselenylmethyl-substituted 1-Phenylethyl Carbenium Ions
Source: Molecules. 2013 Sep 25;18(10):11705–11. doi: 10.3390/molecules181011705 (PMC6270508; doi:10.3390/molecules181011705)
Supplement: Supplementary file 1 [file molecules-18-11705-s001.docx]

Supplementary materials

**Figure S1.** β-selenyl phenylethyl cation -**12 endo**.


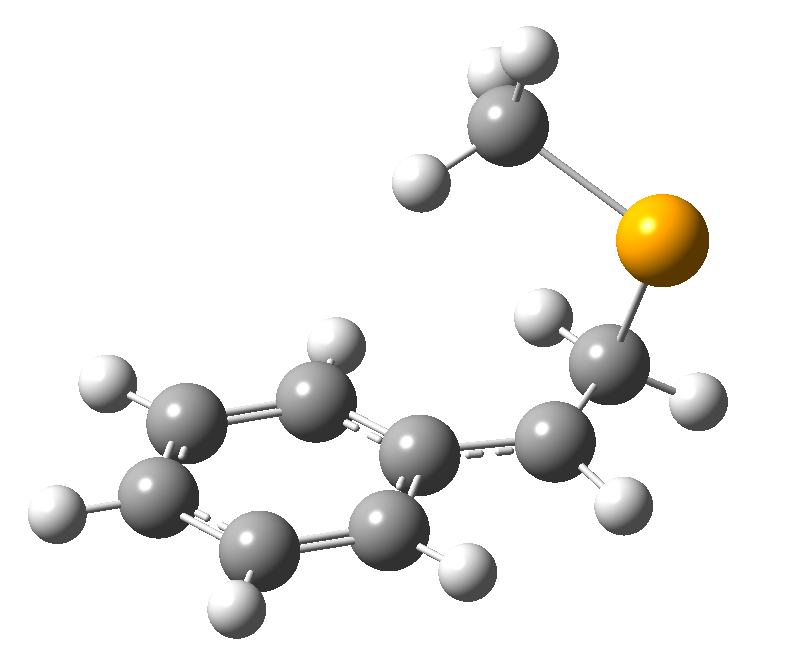


Symbol X Y Z

Se -2.1634830 -0.1281190 -0.2982760

C -0.2224650 -1.0187080 0.6954520

C -1.2664200 -0.3835290 1.4853690

H -1.8941390 -1.0395930 2.0802920

H -0.3840260 -2.0649170 0.4564770

H -1.0647330 0.5674090 1.9651400

C 1.0570470 -0.4786940 0.3435140

C 1.8989160 -1.2476260 -0.4957640

C 1.5352630 0.7536030 0.8497580

C 3.1672650 -0.7981860 -0.8201610

H 1.5430800 -2.1959290 -0.8831620

C 2.8085040 1.1927150 0.5246170

H 0.9273720 1.3486930 1.5202630

C 3.6223510 0.4220520 -0.3116810

H 3.8068880 -1.3926260 -1.4606880

H 3.1771820 2.1283510 0.9266930

H 4.6178980 0.7706790 -0.5606770

C -1.4558230 1.6308330 -0.7993280

H -0.3703890 1.5833920 -0.8863520

H -1.8953570 1.8407500 -1.7744740

H -1.7731730 2.3750790 -0.0727820

1\1\GINC-EDWARD045\FOpt\RB3LYP\6-311++G(d,p)\C9H11Se1(1+)\ROOT\13-Oct2012-\0\\#B3LYP/6-311++G** opt(calcfc) freq=noraman\\\1,1\Se,2.0173360169,-0.3128230372,-0.3932707778\C,0.171218225,0.2747888154,0.9464148733 \C,1.1088295741,-0.7640064464, 1.3452331626\H,1.7944400122,-0.5182166692,2.1501618258\ H,0.4642508607, 1.2912950561, 1.188512581\H,0.7808326834, -1.7970926275,1.3623112173\C,-1.1538831008, 0.1035210673, 0.4293084798\C,-1.8734746536,1.258737016,0.0393684915\C,-1.7909354258,-1.1582359134,

0.3540485556\C,-3.1773377571,1.1545505863,-0.4134270001\H,-1.3953737105, 2.2302147428, 0.099587419\C,-3.0983878602,-1.2523179854,-0.0947227129\H,-1.2779200214, -2.0562721185, 0.6758961796\C,-3.7900451698,-0.100247886,-0.4820288527\H,-3.7227464099,2.0426104467,-0.7077567954\H,-3.5881081638,-2.2174549999,-0.13582079\H,-4.8131230861,-0.1808192128,-0.8305586061\C,1.1168487944,-1.5713868342,-1.597799562\H,0.0478625763,-1.3602472333,-1.6238669332\H,1.5545569148,-1.3811080642,-2.5777847186\H,1.322027901,-2.5918481026,-1.2830915367\\Version=AM64L-G09RevB.01\State=1-A\HF=-2750.9567553\RMSD=5.121e-09\RMSF=3.033e-06\Dipole=-0.1659825,-0.2920708,0.1994424\ Quadrupole=8.8103448,

-2.4971341,-6.3132107,-2.4255585,2.4379362,1.1645868\PG=C01 [X(C9H11Se1)]\\@

**Figure S2.** β-selenyl phenylethyl cation **12-exo**


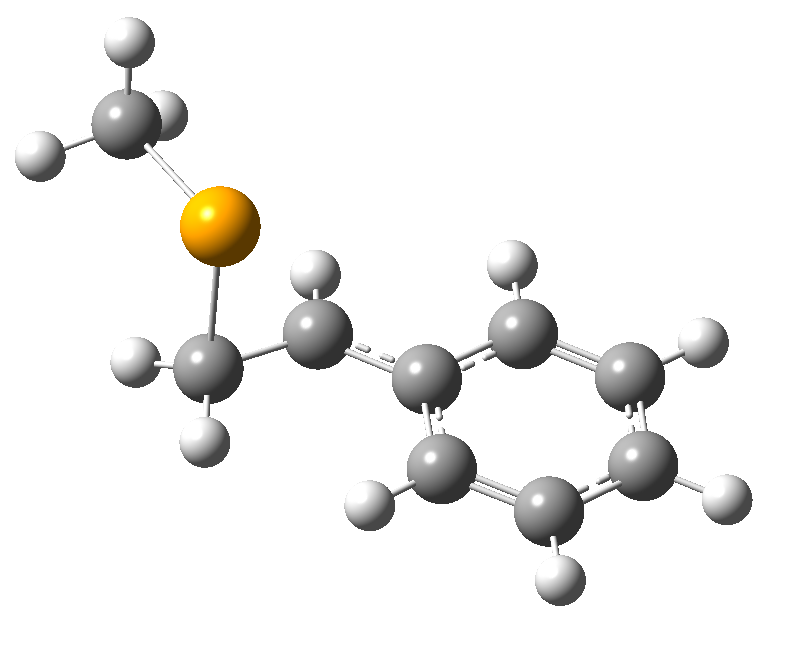


Symbol X Y Z

Se 1.9201140 -0.2585790 -0.5180620

C 0.1275570 0.3448480 0.9126890

C 1.0805960 -0.7005250 1.2535700

H 1.7840410 -0.4835100 2.0509540

H 0.4232150 1.3586030 1.1639890

H 0.7411110 -1.7299790 1.2557150

C -1.2069950 0.1762480 0.4276020

C -1.9736720 1.3384670 0.1712740

C -1.8016020 -1.0943340 0.2396130

C -3.2877960 1.2336590 -0.2511690

H -1.5254410 2.3157080 0.3132710

C -3.1143090 -1.1898920 -0.1865290

H -1.2426980 -2.0016780 0.4336150

C -3.8575730 -0.0292370 -0.4325910

H -3.8714830 2.1260870 -0.4395960

H -3.5693170 -2.1628490 -0.3247700

H -4.8861120 -0.1122050 -0.7641130

C 3.3655560 0.9308890 0.0969600

H 4.0430210 0.3702850 0.7372800

H 3.8770880 1.2529820 -0.8097850

H 2.9521230 1.7975180 0.6090360

NBO

1\1\GINC-EDWARD047\SP\RB3LYP\6-311++G(d,p)\C9H11Se1(1+)\ROOT\07-Oct-2012\ 0\\#B3LYP/6-311++G** Pop=NBOread\\\1,1\Se,0,1.920114,-0.258579,-0.518062\C,0,0.127557, 0.344848,0.912689\C,0,1.080596,-0.700525,1.25357\H, 0,1.784041,-0.48351, 2.050954\ H,0,0.423215,1.358603,1.163989\H,0,0.741111,-1.729979,1.255715\C,0,-1.206995, 0.176248, 0.427602\C,0,-1.973672,1.338467,0.171274\C,0,-1.801602,-1.094334,0.239613\C,0,-3.287796, 1.233659,-0.251169\H,0,-1.525441,2.315708,0.313271\C,0,-3.114309,-1.189892,-0.186529\H,

0,-1.242698,-2.001678,0.433615\C,0,-3.857573,-0.029237,-0.432591\H,0,-3.871483,2.126087,

-0.439596\H,0,-3.569317,-2.162849,-0.32477\H,0,-4.886112,-0.112205,-0.764113\C,0,3.365556, 0.930889,0.09696\H,0,4.043021,0.370285,0.73728\H,0,3.877088,1.252982,-0.809785\H,0,2.952123,

1.797518,0.609036\\Version=AM64L-G09RevB.01\State=1-A\HF=-2750.9559861\RMSD=3.821e-09\ Dipole=0.3763162,0.305088,0.6350613\Quadrupole=16.8960795,-5.0464475,-11.849632, 2.3315173,3.7925232,-0.3935646\PG=C01 [X(C9H11Se1)]\\@

**Figure S3.** β-selenyl 4-aminophenylethyl cation **10-exo**.


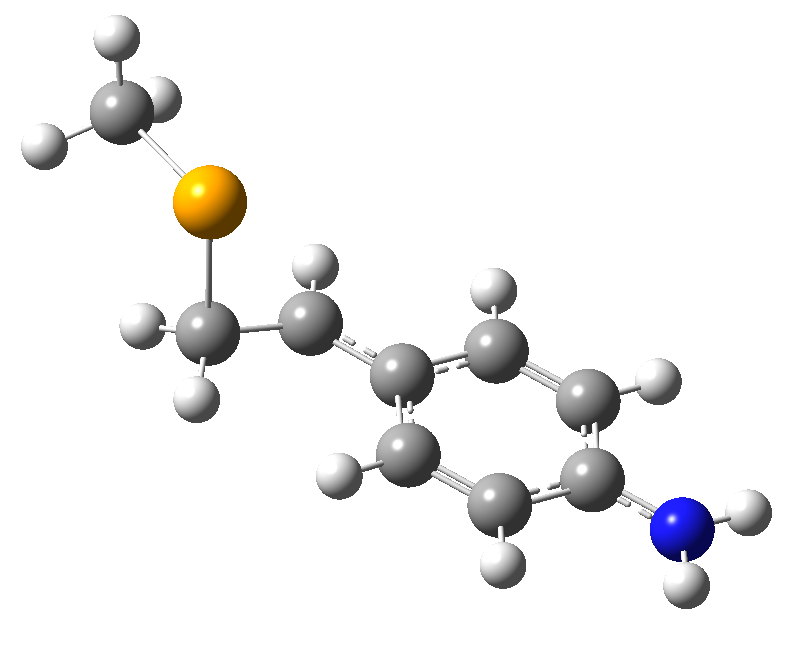


Symbol X Y Z

Se 2.4770840 -0.3831780 -0.4983520

C 0.4082940 0.6410910 0.8305080

C 1.4525550 -0.3026170 1.2313260

H 2.1300420 0.0856900 1.9886480

H 0.7136490 1.6827000 0.7851090

H 1.1276810 -1.3062650 1.4870160

C -0.9074230 0.3619210 0.4830730

C -1.7596230 1.4359980 0.0622950

C -1.4813210 -0.9507720 0.5372420

C -3.0667090 1.2297690 -0.2666000

H -1.3475720 2.4374840 0.0084870

C -2.7857830 -1.1683970 0.2066210

H -0.8757400 -1.7934020 0.8463250

C -3.6183030 -0.0831140 -0.2045310

H -3.6946050 2.0567230 -0.5773730

H -3.2049440 -2.1668610 0.2550180

N -4.9002740 -0.2991350 -0.5258400

H -5.3053330 -1.2222740 -0.4857770

H -5.5038820 0.4548470 -0.8176900

C 3.9737310 0.8104430 -0.0345640

H 4.5209150 0.4039710 0.8135500

H 4.6129360 0.8252860 -0.9172820

H 3.6153920 1.8181520 0.1666060

1\1\GINC-EDWARD047\FOpt\RB3LYP\6-311++G(d,p)\C9H12N1Se1(1+)\ROOT\07-Oct-2012\0\\ #B3LYP/6-311++G** opt(calcfc) freq=noraman\\\1,1\Se,1.0708167027,-0.9397786506,

-0.7504606052\C,-0.7391179094,0.1790165386,0.8518254851\C,0.1187948374,-0.9944542139, 1.0211490464\H,0.8739188516,-0.879911506,1.7957305962\H,-0.2452738259,1.1390263318, 0.9732858353\H,-0.3823637891,-1.952798455,1.1141367289\C,-2.0913391411,0.2097273156, 0.5350998 098\C,-2.7366817344,1.4782770987,0.3583113198\C,-2.8994577059,-0.9655353722, 0.3896472595\C,-4.0663530164,1.5745304024,0.0721531855\H,-2.1457561093, 2.3818437427, 0.4584626344\C,-4.2287243072,-0.8804520046,0.0996407374\H,-2.4555048985, -1.9455415506, 0.511212314\C,-4.8524252225,0.3936204247,-0.0668209478\ H,-4.5351935658, 2.5437129236,

-0.0525781557\H,-4.8261883479,-1.7788267337,-0.0048974526\N,-6.1590936519, 0.4744987265,-0. 348656014\H,-6.7287960742,-0.3520896074,-0.4489266304\H,-6.6173163836, 1.3653189998, -0.468988613\C,2.774535377,-0.140614299,-0.1686870386\H,3.2549581645,-0.787362906, 0.5626390541\H,3.3851252423,-0.0795775108,-1.0694922235\H,2.6158109075, 0.8609222052,0.2263884744\\Version=AM64L-G09 RevB.01\State=1-A\HF=-2806.3573071\ RMSD=3.384e-09\RMSF=1.779e-06\Dipole=-2.1249735,0.6516351,0.5538201\ Quadrupole=33.3368513,-11.8067305,-21.5301208,-4.6803264,4.1897792,-0.6064713\PG=C01 [X(C9H12N1Se1)]\\@

NBO

1\1\GINC-EDWARD029\SP\RB3LYP\6-311++G(d,p)\C9H12N1Se1(1+)\ROOT\07-Oct-2012\0\\ #B3LYP/6-311++G** Pop=NBOread\\\1,1\Se,0,2.477084,-0.383178,-0.498352\C,0,0.408294, 0.641091,0.830508\C,0,1.452555,-0.302617,1.231326\H,0,2.130042,0.08569, 1.988648\ H,0,0.713649,1.6827,0.785109\H,0,1.127681,-1.306265,1.487016\C,0,-0.907423,0.361921, 0.483073\C,0,-1.759623,1.435998,0.062295\C,0,-1.481321,-0.950772,0.537242\C,0,-3.066709, 1.229769,-0.2666\H,0,-1.347572,2.437484,0.008487\C,0,-2.785783,-1.168397,0.206621\ H,0,

-0.87574,-1.793402,0.846325\C,0,-3.618303,-0.083114,-0.204531\H,0,-3.694605,2.056723,

-0.577373\H,0,-3.204944,-2.166861,0.255018\N,0,-4.900274,-0.299135, -0.52584 \H,0,-5.305333,

-1.222274,-0.485777\H,0,-5.503882,0.454847,-0.81769\C,0,3.973731,0.810443,-0.034564\ H,0,4.520915,0.403971,0.81355\H,0,4.612936,0.825286,-0.917282\H,0,3.615392,1.81815

2,0.166606\\Version=AM64L-G09RevB.01\State=1-A\HF=-2806.3573071\RMSD=6.947e-09\ Dipole=-2.2352442,0.3293854,0.3769138\Quadrupole=32.8818873,-12.1493359,-20.7325514, 4.8427899,6.5381188,-1.2006937\PG=C01 [X(C9H12N1Se1)]\\@

**Figure S4.** β-selenyl 4-hydroxyphenylethyl cation **11-exo**


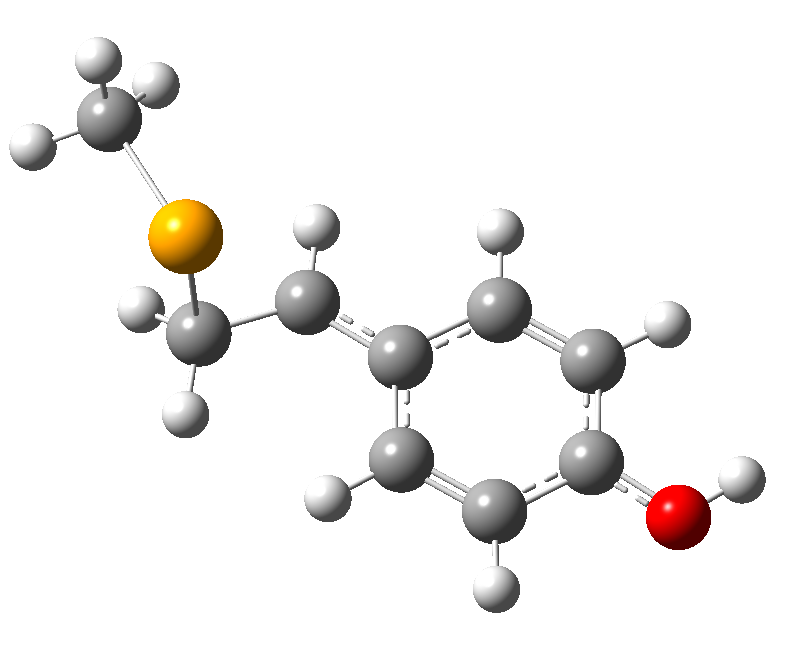


Symbol X Y Z

Se 2.3975800 -0.3350390 -0.5263390

C 0.4593280 0.5205550 0.8991820

C 1.4740240 -0.4658570 1.2548740

H 2.1749260 -0.1500260 2.0229830

H 0.7703700 1.5589690 0.9635730

H 1.1443010 -1.4874230 1.4116300

C -0.8682400 0.2863140 0.5094860

C -1.6833530 1.4024580 0.1594650

C -1.4602080 -1.0122040 0.4764630

C -3.0008160 1.2429320 -0.1914800

H -1.2513250 2.3967850 0.1747080

C -2.7728450 -1.1796350 0.1237310

H -0.8754780 -1.8856320 0.7362610

C -3.5574320 -0.0530970 -0.2138480

H -3.6116000 2.1009430 -0.4503290

H -3.2362540 -2.1576740 0.0990500

O -4.8236790 -0.2921370 -0.5439150

H -5.3065380 0.5164940 -0.7631170

C 3.8791540 0.8623990 -0.0266040

H 4.4973120 0.3816520 0.7286500

H 4.4505640 1.0096930 -0.9429210

H 3.4977790 1.8214550 0.3187520

1\1\GINC-EDWARD034\FOpt\RB3LYP\6-311++G(d,p)\C9H11O1Se1(1+)\ROOT\07-Oct-2012\ 0\\#B3LYP/6-311++G** opt(calcfc) freq=noraman\\\1,1\Se,1.0082817952,-0.8939603442, -0.7585036283\C,-0.6939297863,0.0574416351,0.8902182795\C,0.127227768,-1.1403648396, 1.0319756587\H,0.8963435265,-1.0842307615,1.7976876404\ H,-0.1918296664, 0.9983718444, 1.0945274215\H,-0.383809508,-2.0971406157,1.04949484\C,-2.0523280713,0.1329393857, 0.546384622\C,-2.6530217139,1.4196131409,0.4185574259\C,-2.8781797362,-1.0146016501, 0.3494460229\C,-3.9863826048,1.5595440325,0.1237914104\H,-2.0417965733,2.3037923137, 0.5606762325\C,-4.2083368019,-0.8829258329,0.0514308295\H,-2.4606744862, -2.0095986318, 0.4386169965\C,-4.7767560754,0.4063103442,-0.0642900222\H,-4.4323346056,2.5441411795, 0.0347007234\H,-4.8475814812,-1.7441087817, -0.095703733\O,-6.0741666246,0.4564709135,

-0.3535578903\H,-6.4026684171,1.3636441268,-0.4184443836\C,2.7017701316,-0.0804495465,

-0.1677799917\H,3.2397780122,-0.7801789118,0.4682807131\H,3.264579431,0.1065112294,-1.0821824566\H,2.5170730878,0.8635151703,0.3414 587895\\Version=AM64L-G09RevB.01\ State=1-A\HF=-2826.2130895\RMSD=9.043e-09\RMSF=6.456e-06\Dipole=-0.1934456,0.9271449, 0.7252041\Quadrupole=20.7322223,-5.0745975,-15.6576248,-9.0205504,1.9427964,-0.7299649\ PG=C0 1 [X(C9H11O1Se1)]\\@

NBO

1\1\GINC-EDWARD024\SP\RB3LYP\6-311++G(d,p)\C9H11O1Se1(1+)\ROOT\07-Oct-2012\0\\ #B3LYP/6-311++G** Pop=NBOread\\\1,1\Se,0,2.39758,-0.335039,-0.526339\C,0,0.459328, 0.520555,0.899182\C,0,1.474024,-0.465857,1.254874\H,0,2.174926,-0.150026,2.022983 \H,0,0.77037,1.558969,0.963573\H,0,1.144301,-1.487423,1.41163\C,0,-0.86824,0.286314, 0.509486\C,0,-1.683353,1.402458,0.159465\C,0,-1.460208,-1.012204,0.476463\C,0,-3.000816, 1.242932,-0.19148\H,0,-1.251325,2.396785,0.174708\C,0,-2.772845,-1.179635,0.123731\H,0,

-0.875478,-1.885632,0.736261\C,0,-3.557432,-0.053097,-0.213848\H,0,-3.6116,2.100943,-0.450329\ H,0,-3.236254,-2.157674,0.09905\O,0, -4.823679,-0.292137,-0.543915\H,0,-5.306538,0.516494,

-0.763117\C,0,3.879154,0.862399,-0.026604\H,0,4.497312,0.381652,0.72865\H,0,4.450564,

1.009693,-0.942921\H,0,3.497779,1.821455,0.318752\\Version=AM64L-G09RevB.01\ State=1-A\HF=-2826.2130895\RMSD=6.381e-09\Dipole=-0.3993535,0.9740981,0.560881\ Quadrupole= 22.7361826,-7.8030866,-14.9330961,-3.1070318,4.7889399,-1.7462708\PG=C01 [X(C9H11O1Se1)]\\@

**Figure S5.** β-selenyl 4-nitrophenylethyl cation **13-exo**.


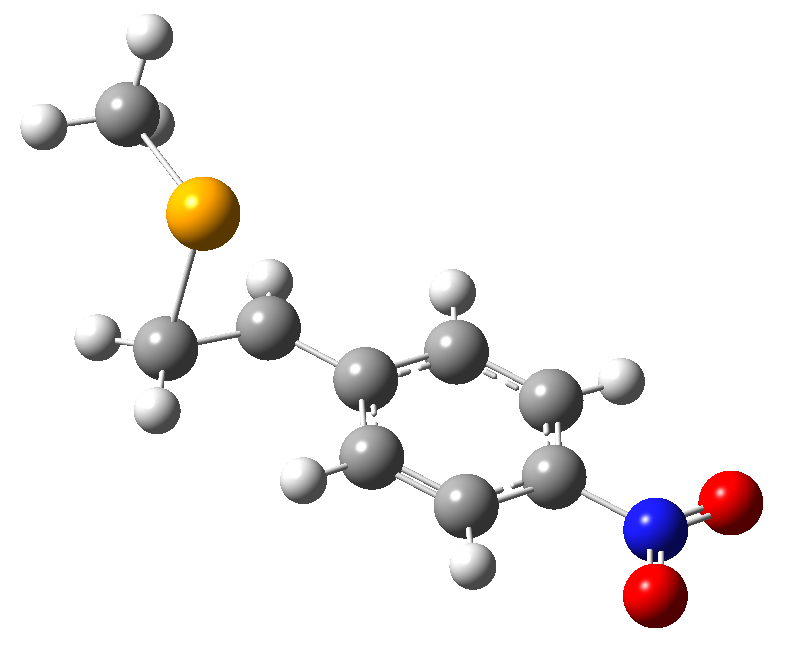


Symbol X Y Z

Se 2.8194420 -0.2419480 -0.6108060

C 1.3226320 0.2976880 0.9728250

C 2.2506590 -0.7922420 1.2371330

H 3.0529520 -0.6160270 1.9452940

H 1.6484040 1.2886100 1.2713650

H 1.8853190 -1.8123790 1.2318560

C -0.0797150 0.1716250 0.6196730

C -0.8313260 1.3546090 0.4679470

C -0.7165950 -1.0764300 0.4678890

C -2.1852070 1.2984840 0.1747290

H -0.3514820 2.3195380 0.5864010

C -2.0688540 -1.1401350 0.1710210

H -0.1698590 -2.0033440 0.5888260

C -2.7772710 0.0493590 0.0288700

H -2.7821950 2.1930110 0.0596790

H -2.5820850 -2.0850250 0.0540740

N -4.2352150 -0.0202900 -0.2896230

O -4.8257080 1.0402680 -0.3996880

O -4.7187110 -1.1323700 -0.4156680

C 4.3505960 0.9150210 -0.1585740

H 5.1397370 0.2982320 0.2659890

H 4.6701820 1.3544670 -1.1029830

H 4.0403500 1.7001290 0.5280570

1\1\GINC-EDWARD046\FOpt\RB3LYP\6-311++G(d,p)\C9H10N1O2Se1(1+)\ROOT\07-

Oct-2012\0\\#B3LYP/6-311++G** opt(calcfc) freq=noraman\\\1,1\Se,0.7786765578,

-0.8484661118,-0.6360063584\C,-0.6153044586,0.0342178308,0.8862356152\C, 0.1185370269,

-1.1737011998,1.2347299831\H,0.9357749805,-1.085432329,1.9422253551\H,-0.1314891684, 0.9755474236,1.1249824148\H,-0.4104328855,-2.1178038569,1.289881349\C,-2.0176121105, 0.1176939079,0.5205135607\C,-2.5625802722,1.3956029299,0.2810355761\C,-2.8514196006,

-1.0157027775,0.4400672795\C,-3.9057900996,1.5439011996,-0.028667672\H,-1.9303143809, 2.2740264046,0.344140303\C,-4.1942666173,-0.8753732953,0.1272373436\H,-2.4660239335,

-2.0099102164,0.6291103021\C,-4.6956021234,0.4024626029,-0.1024452556\H,-4.3461272923, 2.5149038312,-0.2105107906\H,-4.8561529761,-1.7284771743,0.0638276313\N,-6.1435657385, 0.5529136451,-0.4380883651\O,-6.5500644302,1.6865706024,-0.6256041541\O,-6.8037319859

,-0.4702236888,-0.4993003832\C,2.4780442473,0.0679411058,-0.2361647344\H,3.1524404065,

-0.6408462466,0.239479617\H,2.8700828046,0.3850927839, -1.2020204699\H,2.2988501499, 0.9372367284,0.393377253\\Version=AM64L-G 09RevB.01\State=1-A\HF=-2955.5017382\ RMSD=6.610e-09\RMSF=6.253e-06\Dipole=4.7102256,-0.388065,1.0370083\ Quadrupole = 9.3249257, -2.5046853,-6.8202404,0.1674292,-0.4758298,-0.9376926\PG=C01 [X(C9H10N1O2Se1)]\\@

NBO

1\1\GINC-EDWARD028\SP\RB3LYP\6-311++G(d,p)\C9H10N1O2Se1(1+)\ROOT\07-Oct-2012\0 \\#B3LYP/6-311++G** Pop=NBOread\\\1,1\Se,0,2.819442,-0.241948,-0.610806\C,0,1.322632, 0.297688,0.972825\C,0,2.250659,-0.792242,1.237133\H,0,3.052952,0.616027,1.945294\H,0, 1.648404, 1.28861,1.271365\ H,0,1.885319,-1.812379,1.231856\C,0,-0.079715,0.171625, 0.619673\C,0,-0.831326,1.354609,0.467947\C,0,-0.716595,-1.07643,0.467889\C,0,-2.185207,

1.298484,0.174729\H,0,-0.351482,2.319538,0.586401\C,0,-2.068854,-1.140135,0.171021\ H,0,

-0.169859,-2.003344,0.588826\C,0,-2.777271,0.049359,0.02887\H,0,-2.782195,2.193011, 0.059679\H,0,-2.582085,-2.085025,0.054074\N,0,-4.235215,-0.02029,-0.289623\O,0,-4.825708, 1.040268,-0.399688\O,0,-4.718711,-1.13237,-0.415668\C,0,4.350596,0.915021,-0.158574\H,0,5.1397

37,0.298232,0.265989\H,0,4.670182,1.354467,-1.102983\H,0,4.04035,1.700129,0.528057\\ Version=AM64L-G09RevB.01\State=1-A\HF=-2955.5017382\RMSD=5.925e-09\Dipole=4.7251565, 0.3298629,0.988065\Quadrupole=8.9361727,-2.0151894,-6.9209832,2.0963801,-0.421774,

-0.7242696\PG=C01 [X(C9H10N1O2Se1)]\\@
